# Dental service sector and patient-reported oral health outcomes: Modification by trust in dentists

**DOI:** 10.3389/fpubh.2023.1090911

**Published:** 2023-03-15

**Authors:** Youngha Song, Pedro Santiago, Rahul Nair, Hyun-Jae Cho, David Brennan

**Affiliations:** ^1^Department of Preventive and Social Dentistry, School of Dentistry, Seoul National University, Seoul, Republic of Korea; ^2^Dental Research Institute, Seoul National University, Seoul, Republic of Korea; ^3^Australian Research Centre for Population Oral Health, The University of Adelaide, Adelaide, SA, Australia; ^4^Quality and Safety of Oral Health Care Radboud UMC, Nijmegen, Netherlands

**Keywords:** oral health, patient reported outcome measures, dental care, trust, South Australia

## Abstract

**Objective:**

The study aimed to examine the association between patient-reported oral health outcomes and the dental service sector and trust in dentists. The possible interaction effect of trust on this association was also explored.

**Methods:**

Randomly selected adults aged over 18 years living in South Australia were surveyed using self-administered questionnaires. The outcome variables were self-rated dental health and the evaluation outcome of the Oral Health Impact Profile. The dental service sector and the Dentist Trust Scale were included in bivariate and adjusted analyses with sociodemographic covariates.

**Results:**

Data from 4,027 respondents were analyzed. Unadjusted analysis showed that poor dental health and oral health impact were associated with sociodemographic characteristics, including lower income/education, public dental service, and lower trust in dentists (*p* < 0.01). Adjusted associations were similarly maintained (*p* < 0.05) but attenuated with the loss of statistical significance, mainly in the trust tertiles. Lower trust in dentists in the private sector had an interaction effect, with a higher prevalence ratio of oral health impact (prevalence ratio = 1.51; 95% confidence interval, 1.06–2.14; *p* < 0.05).

**Conclusion:**

Patient-reported oral health outcomes were associated with sociodemographic characteristics, the dental service sector, and trust in dentists.

**Implications for public health:**

The inequality of oral health outcomes between dental service sectors needs to be addressed both independently and in association with covariates including socioeconomic disadvantage.

## 1. Introduction

Health outcomes are based on objective clinical test results rather than on the patients' own measures (1). However, the paradigm has shifted to the biopsychosocial model of health where subjective indicators—patient-reported outcomes (PROs) (2)—complementarily or primarily evaluate healthcare practices (3). The rationale for adopting patients' perspectives on measuring health outcomes is in line with patient-centered care, one of the aims for the quality of care (4). PROs refer to “any report coming directly from patients, without interpretation by physicians or others” (2) sharing the core concept with “person-reported outcome” or “self-rated health” (1). Dentistry has also developed and implemented context-/disease-specific PROs, as well as perceived oral health (5). Patient-reported oral health outcomes are commonly assessed using self-rated dental health (6) and oral health-related quality of life (OHRQoL) such as the Oral Health Impact Profile (7).

Clinical encounters remain an essential component in the healthcare system whether in terms of face-to-face practice or online distant consultation (8). Provider–patient relationships are at the center of clinical healthcare (4), which also applies to dentistry (9). Given that normative patterns of patient-centeredness have led to the basis of “relationship-centered care” (10), the assessment of health with PROs should incorporate variables of provider–patient relationships as a potential determinant. To provide more context on oral health outcomes, favorable dentist–patient relationships (DPRs) are empirically associated with better OHRQoL (11, 12). Although it is difficult to operationalize the construct of DPR (13), trust in dentists (14, 15) has been acknowledged as a salient contribution factor to establish a therapeutic relationship along with satisfaction, dental fear, communication, and control at dental encounters (12).

Oral healthcare is generally provided through two dental service sectors: public and private care (16). In Australia, based on the latest national survey, the majority of the adult population (81.8%) made their last dental visit to private practices for the limited eligibility with means tests and long waiting lists (17). Despite the relatively small portion of dental healthcare, public services have been shown to be associated with unfavorable access to services and poor oral health outcomes in significant measures (18, 19). The public dental sector is more likely to have problem-oriented services than preventive/maintenance care (18), leading to a higher prevalence of dental caries and periodontal disease (20). However, the inequity of oral health in different dental service sectors has been largely studied with a focus on clinical outcomes, setting aside patients' perceptions. Furthermore, the relationship between dentists and patients has not been sufficiently considered in this disparity.

Derived from the gap in previous research findings, this study aimed to examine the association of patient-reported oral health outcomes with the dental service sector and trust in dentists, a representative variable of DPR. By extension, we aimed to assess whether trust in dentists has an interaction effect with the dental service sector on the association of oral health outcomes. The main hypothesis to test was that those in public dental services with lower trust in dentists were more likely to have poor patient-reported oral health outcomes. We compared the differences in PROs in oral health between private and public dental care, allowing for sociodemographic characteristics and possible modification of this DPR variable.

## 2. Materials and methods

A total of 12,245 adults aged 18 years or older in South Australia were randomly drawn after stratification by sex and age from the Electoral Roll, a comprehensive sampling frame (21). Data were collected by mailed self-completed questionnaires in 2015–2016, which implied that informed consent was obtained by voluntarily returning the survey forms. The cross-sectional data analyzed in this study were part of the baseline resource for a prospective cohort project for longitudinal changes in oral health outcomes by different determinants (22). This study was approved by the Human Research Ethics Committee of the University of Adelaide (H-288-2011). All the procedures in this study were performed in accordance with the Declaration of Helsinki.

The outcome variables were self-rated dental health (SRDH) and Oral Health Impact Profile (OHIP-14) to assess PROs of oral health. The SRDH is a single item of self-rating global oral health based on the question, “How would you rate your dental health?” with five response levels as follows: excellent, very good, good, poor, and very poor (23). It has been commonly incorporated in population-based surveys (6), with acceptable properties in the validation and predictive capability of clinical outcomes (23). The OHIP-14 is a 14-item scale that captures the perceived oral health impact on a 5-point Likert scale ranging from never to very often (7). The scale has been adequately validated and is widely accepted for assessing OHRQoL (24). For the purpose of analysis in this study, “poor” oral health and oral health “impact” were defined as participants reporting the lowest two response options: either poor or very poor in SRDH and fairly often or very often in any single or multiple items of OHIP-14.

The explanatory variables were the dental service sector and trust in dentists. The dental service sector was dichotomized from the question of where the last dental visit was made with choices of public or private services. Trust in dentists was measured using the Dentist Trust Scale (DTS), an 11-item psychometric scale, on a 5-point Likert scale (from 1 = strongly disagree to 5 = strongly agree) (14). DTS was modified from the original “trust in physicians” scale, and both satisfied construct validity and reliability (Cronbach's α = 0.92 in the current study) (14, 25). The response score for each DTS item was summed (ranging from 11 to 55; higher scores indicated higher trust), and the total score was classified into tertiles as a category variable (lower tertile ranging from 11 to < 38, middle from 38 to < 45, and upper from 45 to 55). Other covariates were included in the analysis to adjust for the demographic and socioeconomic characteristics. Demographic variables were age (categorized as “18–39,” “40–59,” or “≥60” years) and sex (“female” or “male”). Socioeconomic status (SES) was assessed using annual household income (“ < $80,000” or “≥$80,000” in AUD) and the highest level of education completed (“ ≤ year 12 or certificate” or “diploma/degree”).

The collected data were prepared using data cleaning/screening before descriptive statistics and association analyses. Respondents with critical missing values (e.g., SRDH or any item of OHIP-14) and/or the number of missing items >20% in the DTS (≥3 items missing) were filtered out. To prevent acquiescence bias, those with identical responses for all items in the DTS were excluded, considering the inclusion of two reverse-coded items in the scale. Missing values of up to two items in the DTS were imputed using the expectation–maximization algorithm with an iterative maximum-likelihood estimation. Data were weighted by the distribution of age by sex to represent the population estimates of the variables. Descriptive statistics with a frequency table and unadjusted bivariate associations were analyzed for outcomes, explanatory variables, and covariates. Adjusted associations were calculated with prevalence ratios (PRs) using log-binomial regression. Interaction terms between the private dental service sector and levels of trust were included to test possible modifications. SPSS Statistics (version 25.0., IBM Corp., Chicago, IL) was used for all statistical analyses, and a *p*-value of < 0.05 was adopted as the threshold for statistical significance.

## 3. Results

Response data were analyzed from 4,027 respondents after excluding 491 participants who were screened for missing values and unengaged data criteria. The adjusted valid response rate was 40.0%. The sociodemographic characteristics of the study participants were compared with those of the general population census data to check for possible response bias (Supplementary Table S1). The respondents' profile had a close approximation of the population data, with minor differences, mainly in SES. A larger proportion of adults with better SES were sampled in this study with a higher education level of diploma/degree (42.2 vs. 30.0%) and income ranging ≥$80,000 (45.5 vs. 39.8%). Participants were of a slightly higher percentage from the younger age group and private dental sector.

**Figure 1:**
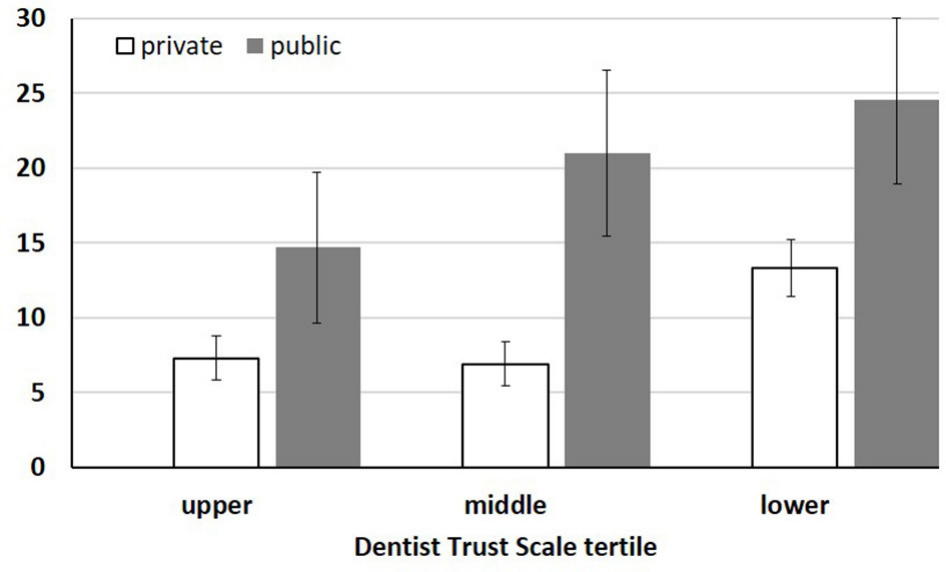
Percentage of ‘poor' to ‘very poor' SRDH (%±SE).

**Figure 2:**
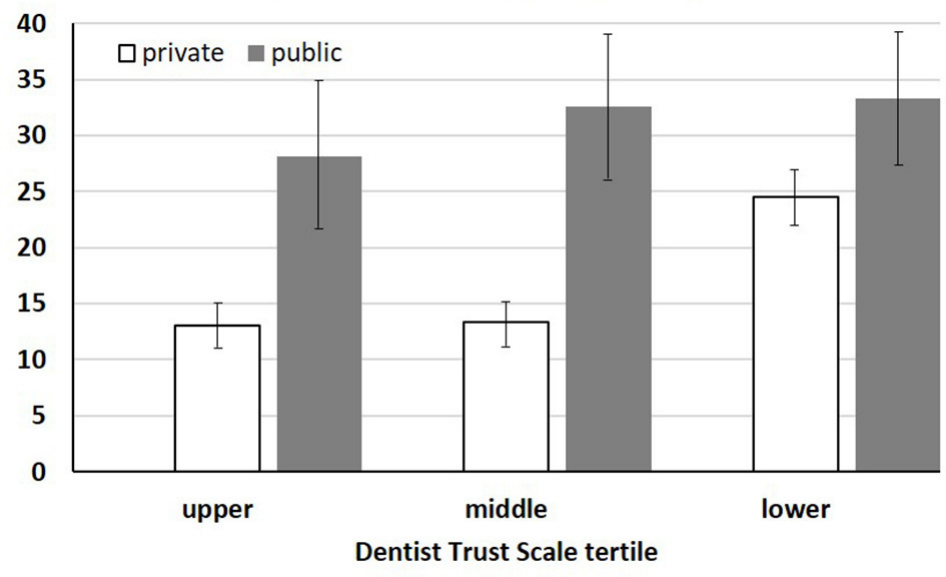
Percentage of OHIP-14 impact (%±SE).

The descriptive statistics and unadjusted associations are presented in Table 1. The mean of the summed DTS scores was statistically different among the age groups and education levels. The older age group and those with lower education levels had higher trust in dentists (*p* < 0.01). The DTS score was slightly higher in the private dental services group, but the difference was not statistically significant (*p* = 0.060). Bivariate association analysis indicated that the prevalence of poor dental health from SRDH and oral health impact from OHIP-14 were associated with all the variables included in the model (*p* < 0.01). The common pattern of both outcome variables was that of a higher prevalence in those with lower income and education, public dental sector, and lower trust in dentists. Figures 1, 2 of stratified percentages confirm the pattern of prevalence in accordance with the dental service sector and DTS tertile. Regarding the prevalence of SRDH, older adults and individuals of the male sex were more likely to report poor oral health. In contrast, the middle-aged group and female sex showed a higher prevalence of oral health impact in OHIP-14. The overall prevalence of poor dental health and oral health impacts was 10.9 and 19.2%, respectively.

**Table 1:** Descriptive statistics and unadjusted/adjusted associations of oral health outcomes.

	**Distribution**	**DTS**	**SRDH**		**OHIP-14**	
	***n*** **(valid %)**	**Mean (SE)**	**%** ^a^	**PR**^b^ **(95% CI)**	**%** ^c^	**PR**^b^ **(95% CI)**
**Age**		**	**		**	
18–39	1,535 (38.1)	40.0 (0.20)	8.3	Ref.	16.5	Ref.
40–59	1,386 (34.4)	39.8 (0.21)	11.9	1.41^**^ (1.13–1.76)	22.0	1.37^**^ (1.18–1.60)
≥60	1,106 (27.5)	43.4 (0.25)	13.1	1.40^**^ (1.13–1.74)	19.3	1.07 (0.91–1.25)
**Gender**			**		**	
Women	2,041 (50.7)	40.9 (0.18)	9.0	0.73^**^ (0.61–0.86)	21.3	1.22^**^ (1.08–1.38)
Men	1,986 (49.3)	40.8 (0.18)	12.8	Ref.	17.0	Ref.
**Income**			**		**	
< $80,000	2,010 (54.5)	40.7 (0.18)	14.6	1.86^**^ (1.48–2.33)	25.6	1.92^**^ (1.65–2.24)
≥$80,000	1,675 (45.5)	40.9 (0.19)	6.3	Ref.	11.9	Ref.
**Education**		**	**		**	
≤ Year 12 or certificate	2,302 (57.8)	41.3 (0.17)	14.0	1.59^**^ (1.30–1.95)	22.0	1.19^**^ (1.05–1.36)
Diploma/degree	1,678 (42.2)	40.3 (0.19)	6.7	Ref.	15.3	Ref.
**Dental service sector**			**		**	
Public	592 (14.7)	40.3 (0.34)	20.5	Ref.	31.5	Ref.
Private	3,435 (85.3)	41.0 (0.14)	9.2	0.64^*^ (0.42–0.98)	17.0	0.55^**^ (0.41–0.73)
**Dentist Trust Scale (DTS)**		**	**		**	
Lower tertile	1,382 (34.3)	32.1 (0.12)	15.1	2.04^**^ (1.33–3.11)	25.9	1.25 (0.92–1.70)
Middle tertile	1,354 (33.6)	41.1 (0.05)	9.0	1.52 (0.97–2.38)	16.2	1.24 (0.91–1.69)
Upper tertile	1,291 (32.1)	50.0 (0.10)	8.3	Ref.	15.1	Ref.
**Interaction**						
Private × DTS lower tertile	–	–	–	0.95 (0.58–1.55)	–	1.51^*^ (1.06–2.14)
Private × DTS middle tertile	–	–	–	0.70 (0.41–1.19)	–	0.89 (0.62–1.28)
Total		40.9 (0.13)	10.9		19.2	

^a^reporting “poor” or “very poor”; ^b^prevalence ratio; ^c^ reporting “fairly often” or “very often” on any items; ^*^p < 0.05; ^**^p < 0.01.

After adjusting for all relevant variables included in the multivariable regression, a similar pattern of association was observed in the adjusted PRs (Table 1). The direction of unadjusted associations was maintained but attenuated with the loss of statistical significance in the DTS middle tertile for SRDH, and age of ≥60 years, and DTS middle and lower tertiles for OHIP-14. There was a significant interaction effect between private dental services and DTS lower tertile on the prevalence of oral health impact (*p* < 0.05). The higher PR (1.51, 95% CI 1.06–2.14) indicated that those with lower trust in dentists in the private sector had a much higher prevalence of oral health impact compared to those in the upper tertile of trust in the private sector. The other interaction terms showed adjusted PRs lower than 1.0 but did not reach statistical significance.

## 4. Discussion

The findings of this study showed that poor dental health and oral health impacts were associated with the dental service sector and trust in dentists. In addition to the main effects of the explanatory variables, lower trust modified the relationship between private dental services and oral health impact as an interaction effect. Throughout the analysis, sociodemographic covariates were associated with PROs of oral health in both the unadjusted and adjusted models.

This study reaffirms the social gradient and inequality in oral health (26). Those in worse SES with lower income and education level were more likely to be consistently involved in unfavorable oral health outcomes in both bivariate and multivariable analyses. Regardless of clinical indicators or subjective self-ratings of oral health (26), the pattern has been deeply rooted in the social determinant framework (27). Although social inequalities in oral health have been highlighted over the past few decades, their root causes are still yet to be addressed properly and inveterately (28). From the perspective of the oral healthcare system, inequality also depends on disparities in access to care (18) and its relevant clinical outcomes (20) between the private and public dental service sectors. This study reported a similar finding that public dental service users had a higher prevalence of poor dental health and oral health impacts as subjective oral health outcomes. In particular, the association between oral health outcomes and the dental service sector remained statistically significant after adjusting for SES variables—socioeconomic disadvantages (19). This indicates the need to investigate the role of the dental care delivery system as an independent determinant of oral health outcomes. In addition, trust in dentists was included in the model as a psychosocial factor at the micro level of social dentistry (29). The empirical results of the association between higher trust and better oral health outcomes can support the rationale for a favorable DPR beyond normative suggestions (15).

An incongruent pattern of reporting oral health was found for sex differences. Female participants self-rated their dental health on a better level (9.0 vs. 12.8% in SRDH) but felt more of an oral health impact (21.3 vs. 17.0% in OHIP-14) than their male counterparts. This inconsistency has been consistently presented in a series of population-based surveys (three national surveys conducted between 2004 and 2018) in Australia, where the current study was performed (Supplementary Table S2). With no exception since 2004, sex differences in Australian adults indicated that women have better self-rated dental health but more complaints in specific dental conditions. Cognitive dissonance may occur from conceptual differences between SRDH and OHIP-14, despite their commonality as PROs. The former focused on self-rating global oral health, but the specific referents were taken differently by respondents (30), which contrasts with the latter of the less equivocal multi-item scales based on seven dimensions (7). More specifically, on demographics, the pattern may be derived from the finding that women were likely to perform better oral hygiene behaviors but report more concerns about dental complaints (31). However, opposing results have also been reported, such as Asian American subgroups (32) and Brazilian adolescents (33). The sex difference in PROs of oral health needs to be investigated further in a rigorous systematic search (31).

The interpretation of the interaction effects with adjusted PRs requires caution. Table 1 shows the PRs in a relative frame rather than a subgroup analysis. For example, aside from statistical significance, the PRs (< 1.0) of interaction terms should not be interpreted as those in the lower/middle tertile of trust having a lower prevalence of oral health outcomes in each sector. Instead, the significant PR of the interaction indicates that the negative effect of lower trust leading to an oral health impact is more pronounced in private dental services than in the public sector. As shown in Figure 2, the increase in the prevalence of OHIP-14 by lower trust is far greater in private services, resulting in a much smaller relative difference from the prevalence in the public sector than in any other segment. The possibility of potential confounding or mediation should also be considered. The claim that trust in dentists may be a confounder was dismissed, as the distribution of DTS tertiles in the private and public sectors was not statistically different (*p* = 0.201). For the mediation effect, in addition to the similar distribution, it appeared to be less likely that the association between outcomes and the dental service sectors would remain statistically significant after adjusting for DTS in the model (Table 1).

Figures 1, 2 show the prevalence of PROs and the pattern of how participants report oral health outcomes by dental service sector and DTS tertile levels. If private dental patients have lower trust in dentists, they are likely to report disproportionally worse oral health than those with middle and upper levels of trust (prevalence from upper to lower tertile: 7.3%, 6.9%, and 13.3% for SRDH; 13.0%, 13.4%, and 24.5% for OHIP-14). Compared with the private sector, public dental service users are likely to detect higher trust in dentists in terms of oral health outcomes than those in the middle and lower DTS tertiles. This pattern in the public sector appears clearly for the prevalence of OHIP-14 (28.2, 32.5, and 33.3%), and SRDH also partially supports it with a difference of 3.5% from middle to lower tertiles vs. 6.3% from upper to middle DTS tertiles (14.7, 21.0, and 24.5%).

This study has some limitations. The cross-sectional design can only purport the association of outcomes with explanatory variables, not necessarily causal inferences from the findings. Despite a similar profile to the aforementioned population, study participants might have different characteristics, causing selection bias. For example, the overall prevalence of poor oral health in SRDH was 10.9% in this study, which was considerably lower than that reported in national surveys [23.9% in 2017–2018 (17) and 18.8% in 2010 (34)]. However, this discrepancy may be due to a measurement bias with different rating statements. The two national surveys adopted lower response levels with “fair” and “poor” rather than “poor” and “very poor” options in this study for the definition of poor oral health. Thus, the rating scale should be consistent across studies for comparability in future. Another limitation is the absence of important covariates due to the study topic. Except for sociodemographic variables, oral health behaviors (e.g., tooth brushing and smoking) and dental service variables (e.g., time since/purpose of the last dental visit and perceived dental needs) have been reported to be associated with the dental service sector (18, 35) and oral health outcomes (12, 36). Moreover, adult development may be associated with the acceleration of trust (37), which needs to be considered as a potential covariate in further studies. Nevertheless, this study could provide a dental care system with a more comprehensive understanding of PROs, as multiple measures are recommended to assess different aspects of perceived oral health for dental service planning (38).

The findings of this study have practical implications. First, efforts to tackle inequality in oral health need to command attention in terms of the dental service sector. In addition to socioeconomic disadvantages, the quality of public dental services should also be considered an independent factor. Second, trust in dentists for better DPR may be a determinant of PROs of oral health. In particular, lower trust harshly impacts private dental patients—the majority of dental services provided in Australia—than those in the public sector. Finally, for female adult patients in Australia, a probing single question about global oral health asked by clinicians in dental encounters may lead to missing out on specific dental conditions inadvertently. Considering that women are more likely to experience communication problems with their dentists (31), the clinical implication may be salutary to establish better DPR with female patients.

## 5. Conclusion

Patient-reported oral health outcomes were associated with sociodemographic characteristics, the dental service sector, and trust in dentists. Lower trust in dentists in private dental care had a disproportionately worse effect on oral health impact compared to those with higher trust in the private service sector. The disparity in oral health outcomes between dental service sectors needs to be addressed both independently and in association with covariates, including socioeconomic disadvantages. Trust in dentists should also be established to improve oral health outcomes, particularly for private dental service users with lower levels of trust.

## Data availability statement

The raw data supporting the conclusions of this article will be made available by the authors, without undue reservation.

## Ethics statement

The studies involving human participants were reviewed and approved by the Human Research Ethics Committee of the University of Adelaide. The patients/participants provided their written informed consent to participate in this study.

## Author contributions

YS contributed to the conception of the article, data analysis, interpretation of results, and drafting of the manuscript. PS, RN, and H-JC contributed to the interpretation of results and critical revision of the manuscript. DB contributed to the conception of the article and critical revision of the manuscript. All authors have read and approved the content of the manuscript.
