# Supplementary material for: Dental service sector and patient-reported oral health outcomes: Modification by trust in dentists
Source: Front Public Health. 2023 Mar 15;11:1090911. doi: 10.3389/fpubh.2023.1090911 (PMC10050452; doi:10.3389/fpubh.2023.1090911)
Supplement: Supplementary file 1 [file Table_1.DOCX]

Supplementary material

**Dental service sector and oral health outcomes: modification by trust in dentists**

Youngha Song, Pedro Santiago, Rahul Nair, Hyun-Jae Cho, David Brennan

Table S1. Study participants’ sociodemographic characteristics ………………… 2

Table S2. Percentage of reporting each oral health conditions …………………… 3

**Table S1**. Study participants’ sociodemographic characteristics in percentage and comparison with population data

|  | Data from 2016 Census^a^ (%) | Distribution in the study (95% CI) |
| --- | --- | --- |
| Sex |  |  |
| Female | 50.7^­0^ | 50.7 (49.1-52.2) |
| Male | 49.3^­0^ | 49.3 (47.8-50.9) |
| Age |  |  |
| 18–39 | 33.4^b^ | 38.1 (36.6-39.6) |
| 40–59 | 34.7^­0^ | 34.4 (33.0-35.9) |
| ≥60 | 31.8^­0^ | 27.5 (26.1-28.9) |
| Income^c^ |  |  |
| <$80,000 | 60.2^d^ | 54.5 (52.9-56.1) |
| ≥$80,000 | 39.8^­0^ | 45.5 (43.9-47.1) |
| Education |  |  |
| ≤Year 12 or certificate | 70.0^­0^ | 57.8 (56.3-59.4) |
| Diploma/degree | 30.0^­0^ | 42.2 (40.6-43.7) |
| Dental service sector |  |  |
| Private | 81.8^f^ | 85.3 (84.2-86.4) |

^a^ 2016 Census: South Australia (from the Australian Bureau of Statistics <https://quickstats.censusdata.abs.gov.au/census_services/getproduct/census/2016/quickstat/4>); ^b^ Age 20-39; ^c^ annual household in AUD; ^d^ <$78,000 (<$1,500/week); ^f^ Chrisopoulos S, Luzzi L & Ellershaw A 2019. Dental care. P97-124. In: ARCPOH. Australia’s Oral Health: National Study of Adult Oral Health 2017–18. Adelaide: The University of Adelaide, South Australia.

**Table S2**. Percentage of people reporting each oral health condition in the Australian population (95% CI)

|  |  | ‘Poor’ dental health^a^ | Avoid food^b^ | Toothache^c^ | Appearance^d^ / orofacial pain^e^ | Oral health impact |
| --- | --- | --- | --- | --- | --- | --- |
| NSAOH^f^ 2004-06 | Female | 15.8 (14.7–16.9) | 20.2 (19.1–21.3) | 16.2 (15.1–17.3) | 26.9 (25.7–28.1)^e^ | – |
|  | Male | 17.0 (15.7–18.4) | 14.4 (13.2–15.7) | 14.1 (12.8–15.5) | 18.2 (16.8–19.7)^e^ | – |
| NDTIS^g^ 2010 | Female | 17.4 (15.7–19.3) | 21.2 (19.2–23.2) | 15.8 (14.1–17.7) | 28.5 (26.1–30.5)^d^ | 41.5 (39.2–43.9)^i^ |
|  | Male | 20.4 (18.2–22.9) | 13.0 (11.4–14.8) | 14.8 (13.0–16.8) | 21.9 (19.6–24.5)^d^ | 31.9 (29.5–34.4)^i^ |
| NSAOH 2017-18 | Female | 22.7 (21.3–24.2) | 27.2 (25.8–28.6) | 21.6 (20.4–22.9) | 38.4 (37.0–39.9)^d^ | – |
|  | Male | 25.1 (23.6–26.7) | 20.1 (18.8–21.4) | 18.7 (17.3–20.1) | 31.9 (30.2–33.6)^d^ | – |
| DCOH^h^ 2015-16 | Female | 9.0 (7.8–10.3) | – | – | – | 21.3 (19.6–23.1)^j^ |
|  | Male | 12.8 (11.4–14.4) | – | – | – | 17.0 (15.4–18.7)^j^ |

^a^ Reporting lower two responses on self-rated dental health; ^b^ avoiding foods due to dental problems; ^c^ experiencing toothache; ^d^ uncomfortable about dental appearance; ^e^ experiencing orofacial pain; ^f^ The National Survey of Adult Oral Health; ^g^ The National Dental Telephone Interview Survey; ^h^ Dental Care and Oral Health study for the current manuscript; ^i^ reporting any of oral health conditions in the survey; ^j^ reporting ‘fairly often’ or ‘very often’ on any items in the Oral Health Impact Profile-14
